# Feasibility and Acceptability of Automated Texts to Offer, Screen, and Enroll Patients in a Cancer Clinical Trial Financial Reimbursement Program: Mixed Methods Study

**DOI:** 10.2196/78916

**Published:** 2026-07-09

**Authors:** Ashley E Santaniello, Hena Patel, Sarah Milinski, Mohan Balachandran, Vivian Nguyen, E Paul Wileyto, Robert H Vonderheide, Dana Dornsife, Robert G Johnson, Carmen E Guerra

**Affiliations:** 1 Abramson Cancer Center, University of Pennsylvania, Philadelphia, PA, United States; 2 School of Arts and Sciences, University of Pennsylvania, Philadelphia, PA, United States; 3 Way to Health, Penn Medicine Center for Health Care Transformation and Innovation, University of Pennsylvania, Philadelphia, PA, United States; 4 Sidney Kimmel Medical College, Thomas Jefferson University, Philadelphia, PA, United States; 5 Department of Biostatistics Epidemiology and Informatics, Perelman School of Medicine, University of Pennsylvania, Philadelphia, PA, United States; 6 Department of Medicine, Division of Hematology Oncology, Perelman School of Medicine, University of Pennsylvania, Philadelphia, PA, United States; 7 Lazarex Cancer Foundation, Danville, California, United States; 8 Department of Medicine, Division of General Internal Medicine, Perelman School of Medicine, University of Pennsylvania, 3400 Civic Center Blvd, Philadelphia, PA, 19104, United States, 1-215-662-4000

**Keywords:** cancer, clinical trials, financial toxicity, text-based, mixed methods

## Abstract

**Background:**

Out-of-pocket (OOP) costs pose a significant barrier to participating in cancer clinical trials (CCTs). Financial reimbursement programs (FRPs) that reduce the burden of OOP costs can support participation in CCTs if the information is readily available to participants at the time of enrollment. Prior studies have shown the importance and impact of FRPs, but despite improvements, significant barriers still remain.

**Objective:**

This study was designed to explore the feasibility and acceptability of automated texts designed to offer, screen, and enroll CCT participants in an FRP for OOP travel and lodging-related clinical trial costs.

**Methods:**

This study used a mixed methods approach. Eligible participants were those who consented to participate in a breast, leukemia, or chimeric antigen receptor T cell (CAR-T) trial at the Abramson Cancer Center of the University of Pennsylvania, a National Cancer Institute comprehensive cancer center. Quantitative data were collected through engagement metrics, including text response rates and enrollment rates, as well as patient-reported satisfaction scores. Qualitative data were derived from semistructured interviews. Program enrollment rates were used to determine feasibility, whereas the engagement metrics were used to measure the acceptability of the program. Semistructured interviews were conducted with a subsample of patients who responded to at least one of the FRP texts and agreed to be interviewed to determine the barriers to and facilitators of enrolling in the Improving Patient Access to Cancer Clinical Trials (IMPACT) program via text, perceived advantages and disadvantages of the text messaging program compared to a phone call, and overall feedback on the acceptability of the automated text messaging program.

**Results:**

Quantitative data, including engagement with texts, FRP eligibility screening, and enrollment rates, were collected from all participants who successfully received a text (n=51), and qualitative data were collected from a subsample of participants who agreed to participate in a semistructured interview (n=28) about the text-based program. Participants’ mean age was 58 (SD 12) years, approximately 65% (n=33) of participants were female, 21% (n=11) of participants were Black, and 4% (n=2) of participants were Hispanic or Latino. There was high engagement with texts (n=49, 96.1%) and a high screening rate for FRP eligibility (n=33, 64.7%). Of those who successfully screened, 26 (51%) screened via text. We also saw high overall FRP enrollment rates of those who completed the texts (n=16 of 24 eligible, 66.7%) and high satisfaction (Net Promoter Score=51). The text-based platform streamlined the enrollment process, allowing one-third of patients to complete enrollment independently, without assistance from the FRP coordinator. Reported facilitators for completion of the text conversation included support from the coordinator and introduction of the FRP by CCT teams. Barriers were a lack of communication from CCT teams, patient skepticism about the legitimacy of the texts, and limited program information via text.

**Conclusions:**

Despite the small sample size and single study site, these findings suggest that automated text messaging can be an effective, low-cost, and scalable strategy to increase awareness and streamline enrollment in FRPs.

## Introduction

### Background

Approximately one-half of patients with a cancer diagnosis experience financial toxicity (FT), a term used to describe the negative impact that medical expenses can have on patients and their families [1-4]. Out-of-pocket (OOP) expenses, such as medical copays, travel and lodging costs, and patient and caregiver lost wages, contribute to the high FT experienced by patients with cancer. These costs also pose a significant barrier to participating in cancer clinical trials (CCTs). In 1 study, 48% of patients in phase 1 trials reported monthly OOP costs of at least US $1000 [5]. Patients with cancer experience higher OOP costs compared to other chronic diseases [6] because of a higher frequency of visits related to a trial’s protocol and need for lodging for observation [7], which poses a barrier to CCT enrollment [8].

To address the high OOP costs in CCT and attempt to mitigate the FT associated with CCTs, the Lazarex Cancer Foundation created the Improving Patient Access to Cancer Clinical Trials (IMPACT) Program in 2018. The IMPACT Program is a financial reimbursement program (FRP) for OOP travel and lodging expenses associated with CCT participation. Prior studies have demonstrated that IMPACT sites saw higher rates of patient enrollment in CCTs and increased participation among underrepresented groups, including racial and ethnic minorities and patients from lower socioeconomic backgrounds [9]. However, despite initial positive results, a subsequent study assessing the effectiveness of the program revealed that more than 40% of CCT participants offered the IMPACT program did not enroll, suggesting the presence of persistent barriers [10].

As a result, IMPACT 1.0, a precursor to this study, was launched to better understand the barriers to and facilitators of enrolling in the Lazarex IMPACT program at the Abramson Cancer Center. IMPACT 1.0 involved enrollment in the FRP via a telephone call with an IMPACT coordinator. The initial study uncovered several patient barriers to enrolling in the IMPACT program: the CCT team did not mention the IMPACT FRP to patients, leading to a lack of awareness, and when they did, it was often mid or late in the study, missing opportunities for reimbursement of expenses and difficulties connecting with patients via phone [11]. In addition, the program poses a large administrative burden on the IMPACT team and clinical trial (CT) coordinators. This was particularly due to repeated unsuccessful attempts to contact patients via telephone and email to offer the program, which was not only inefficient but also delayed the enrollment process and potentially discouraged program and CCT participation. These findings highlight the need for a more streamlined, accessible, and timely approach to offering and enrolling patients in the IMPACT FRP.

This study leveraged novel digital technology to address these barriers and evaluate the feasibility and acceptability of using an automated text-based system to systematically offer, screen, and enroll patients in the IMPACT program via Way to Health (W2H), a web-based platform created by the Penn Medicine Center for Health Care Innovation and Penn Center for Health Incentives and Behavioral Economics. Text messaging is increasingly being used for communication between health care sites and patients to coordinate care as well as in medical research where it has been used to remind patients about study tasks [12]. The automated text-messaging system was designed to provide a systematic way of offering patients the IMPACT program. It allowed patients to self-screen for the program’s household income–based eligibility criteria and provide flexibility for enrollment based on patient preference and need by allowing patients to complete the application independently via a text or email link to the application or via an IMPACT coordinator telephone call.

### Aims and Hypotheses

Our objective was to determine the feasibility, patient acceptability, and satisfaction of automated text messaging to offer, screen, and enroll patients in the IMPACT FRP. In addition, we hypothesized that direct texts to patients to offer, screen, and enroll in the FRP would reduce administrative burden on the IMPACT team and Lazarex Cancer Foundation and ensure that all eligible patients, regardless of perceived need, are offered the opportunity to participate in the IMPACT program. A secondary objective was to identify the barriers to and facilitators of using the text-based system and subsequently enrolling in the IMPACT program.

## Methods

### Study Design

This study used a mixed methods approach using an explanatory sequential design. Quantitative data were collected through engagement metrics, including text response rates and enrollment rates, as well as patient-reported satisfaction scores. Program enrollment rates were used to determine feasibility, whereas the engagement metrics were used to measure the acceptability of the program. We then conducted semistructured interviews with a subsample of patients who responded to at least one of the FRP texts and agreed to be interviewed to determine the barriers to and facilitators of enrolling in the IMPACT program via text, perceived advantages and disadvantages of the text messaging program compared to a phone call, and overall feedback on the acceptability of the automated text messaging program. The qualitative data were used to better understand the quantitative data and inform implementation.

### Study Population and Eligibility

Patients eligible to receive texts regarding the FRP consisted of a subgroup of patients enrolled in cancer treatment CTs, specifically breast, leukemia, and chimeric antigen receptor T-cell (CAR-T)–treated neoplasms (lymphoma, glioblastoma, prostate, and pancreatic adenocarcinoma), at the Abramson Cancer Center of the University of Pennsylvania, Philadelphia, between March 26, 2024, and July 31, 2024. The 3 CT teams from which we received patient referrals were chosen by the IMPACT study team based on IMPACT 1.0 referral and enrollment trends and diversity of enrolled patients. Trial teams’ willingness to participate was also taken into account.

### Recruitment

CCT participants were referred by their CT coordinators to the IMPACT study team once weekly, typically at the end of the week. To establish a consistent protocol, we asked CT teams to refer all patients to IMPACT, regardless of perceived or known financial need. Additionally, we asked teams to notify patients about the IMPACT program and upcoming texts as well as provide our study flyer at the time of their CCT informed consent. As early as possible during the following week, the IMPACT coordinator would add patients to a list within the electronic medical record, linked to the W2H platform by a query. The query automatically enrolled patients in the W2H automated text messages and sent them the first text.

The texts (Multimedia Appendix 1) required participants to verify (1) their identity, (2) enrollment or consideration of enrollment in a cancer treatment CT, and (3) that they met FRP income eligibility by texting their household size and reviewing the upper limit of income for a household of that size. If they met the eligibility criteria, then patients were offered enrollment via an online application link sent via text or email or via a telephone call with the IMPACT coordinator (Multimedia Appendix 2).

### Data Collection

Eligible participants were invited to apply to the Lazarex IMPACT FRP. Those who completed the text conversation subsequently received a Net Promoter Score (NPS) survey through text as the last question. Thus, we collected satisfaction data from this smaller subgroup of participants who completed the entire text-based conversation. Those who did not complete the text-based conversation and who participated in an interview completed the NPS survey administered as the last question of the interview process.

Patients who engaged with the automated text messages were invited to participate in an approximately 10-minute semistructured interview via telephone regarding their experience in exchange for a US $25 Consumer Value Store (CVS) Gift Card. Patients were only invited to interview once they met the following conditions: (1) disqualified for the FRP via text and (2) opted out of text conversation (responded “BYE”), (3) successfully received and submitted an application for the FRP or (4) after the conversation closed, following nonresponse.

All patients who were interested in completing the semistructured interview were read a statement of research before completion of the interview. Interviews were conducted by the IMPACT coordinator (AS) between April 2024 and October 2024.

### Data Analysis

Acceptability was defined as the extent to which users considered the automated text appropriate, as measured by engagement rates and satisfaction items, two constructs of the Theoretical Framework for Acceptability [13]. Acceptability of FRP automated texts was assessed by measuring engagement rates with the texts at each point in the text conversation (offering, screening, and enrollment), whereas feasibility was evaluated based on FRP program enrollment rates. Feasibility was defined as the proportion of eligible patients completing enrollment in the IMPACT FRP via the text-based workflow, reflecting the intervention’s practicality and adoptability under real-world constraints.

The NPS was used to measure patient satisfaction with the automated text messages. The NPS survey allowed respondents to rate their likelihood of recommending the text conversation on a scale of 1 to 10, with zero being completely unlikely and 10 being extremely likely [14]. To calculate the NPS, the percentage of detractors is subtracted from the percentage of promoters, where scores of 0 to 6 are detractors, 7 to 8 are passives, and 9 to 10 are promoters. The NPS can fall between −100 and +100 [14]. An NPS of >0 is considered “good,” >50 is considered “excellent,” and >70 is considered “best in class” [15]. Our version of the NPS was as follows: “Using a 0‐10 scale: How likely is it that you would recommend using this text-based platform to a friend or colleague?”

Interviews were recorded and transcribed verbatim and subsequently imported into NVivo 12.0 (QSR International). They were conducted, read, and coded independently by 2 investigators (AS and HP) and then coded jointly using consensus conferences to resolve coding disagreements. Thematic analysis was used to identify themes and subthemes. The interviews were performed and analyzed until thematic saturation—the point at which additional interviews did not yield new themes—was reached [16].

### Ethical Considerations

This study was approved by the University of Pennsylvania’s Institutional Review Board (protocol 854446). All participants who received a text were provided the opportunity to opt out of the text conversation. Interview participants gave informed consent prior to the recorded interview. No waiver of consent was requested or granted for primary data collection or analyses. Secondary analyses performed were conducted under original institutional review board protocol, which allows for these analyses without additional participant consent. All study data were treated as confidential and stored in secure systems only accessible to authorized research team members. Identifying information was removed, and data presented in this manuscript were deidentified, ensuring individual participant identities were protected throughout all phases of the research process. Qualitative interview participants were compensated for their time and insights with a US $25 CVS gift card, as disclosed in both the informed consent process and study protocol.

## Results

### Overview

**Figure 1.**
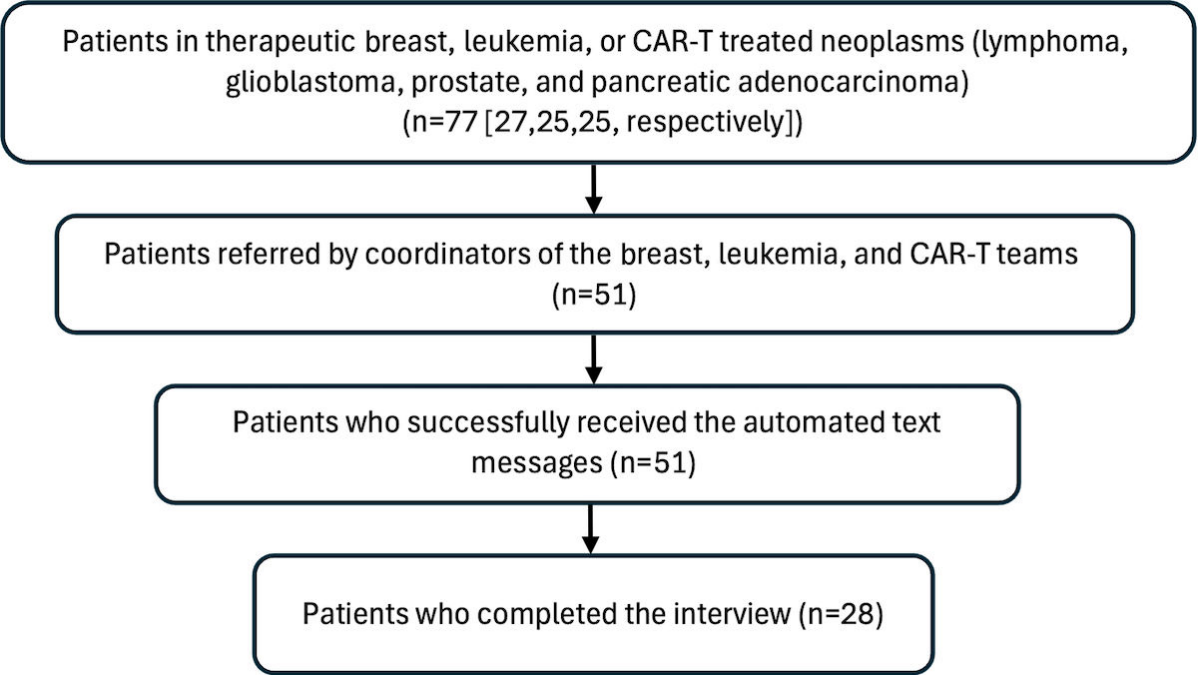
Patient population. CAR-T: chimeric antigen receptor T cell.

As shown in Figure 1, the study sample consisted of 51 CT participants who successfully received a text message regarding the IMPACT Program. Interviewed patients consisted of a convenience subsample of 28 CT participants who successfully received at least one of the IMPACT program study enrollment text messages and consented to the interview.

Table 1 presents the demographic and trial participation data for the sample who received text messages and the subsample of patients who completed interviews. When comparing the 2 samples, the median and average age and representation by sex, race, and ethnicity distribution were similar. The interview sample had slightly higher breast and CAR-T trial participants. The overall sample of patients who received program texts (n=51) had slightly higher leukemia trial participants than the subsample who were interviewed (n=28).

**Table 1.** Patient demographics.

	Received program texts (n=51)	Interviewed (n=28)
Age, mean (SD), median	57.7 (10.3), 58.0	57.8 (11.7), 59.0
Age (years), n (%)		
20‐29	1 (1.9)	0 (0)
30‐39	2 (3.9)	1 (3.6)
40‐49	9 (17.6)	4 (14.3)
50‐59	14 (27.5)	9 (32.1)
60‐69	18 (35.3)	11 (39.3)
70‐79	6 (11.8)	3 (10.7)
80‐89	1 (1.9)	0 (0)
Sex, n (%)		
Female	33 (64.7)	18 (64.3)
Male	18 (35.3)	10 (35.7)
Ethnicity, n (%)		
Not Hispanic or Latino/a	49 (96.1)	27 (96.4)
Race, n (%)		
White	40 (78.4)	22 (78.6)
Black	11 (21.6)	6 (21.4)
Asian	0 (0)	0 (0)
American Indian or Alaska Native	0 (0)	0 (0)
Native Hawaiian or other Pacific Islander	0 (0)	0 (0)
Disease team, n (%)		
Breast	20 (39.2)	12 (42.9)
CAR-T^a^	19 (37.3)	12 (42.9)
Leukemia	12 (23.5)	4 (14.3)

a CAR-T: chimeric antigen receptor T cell.

### Quantitative

#### Engagement

**Figure 2.**
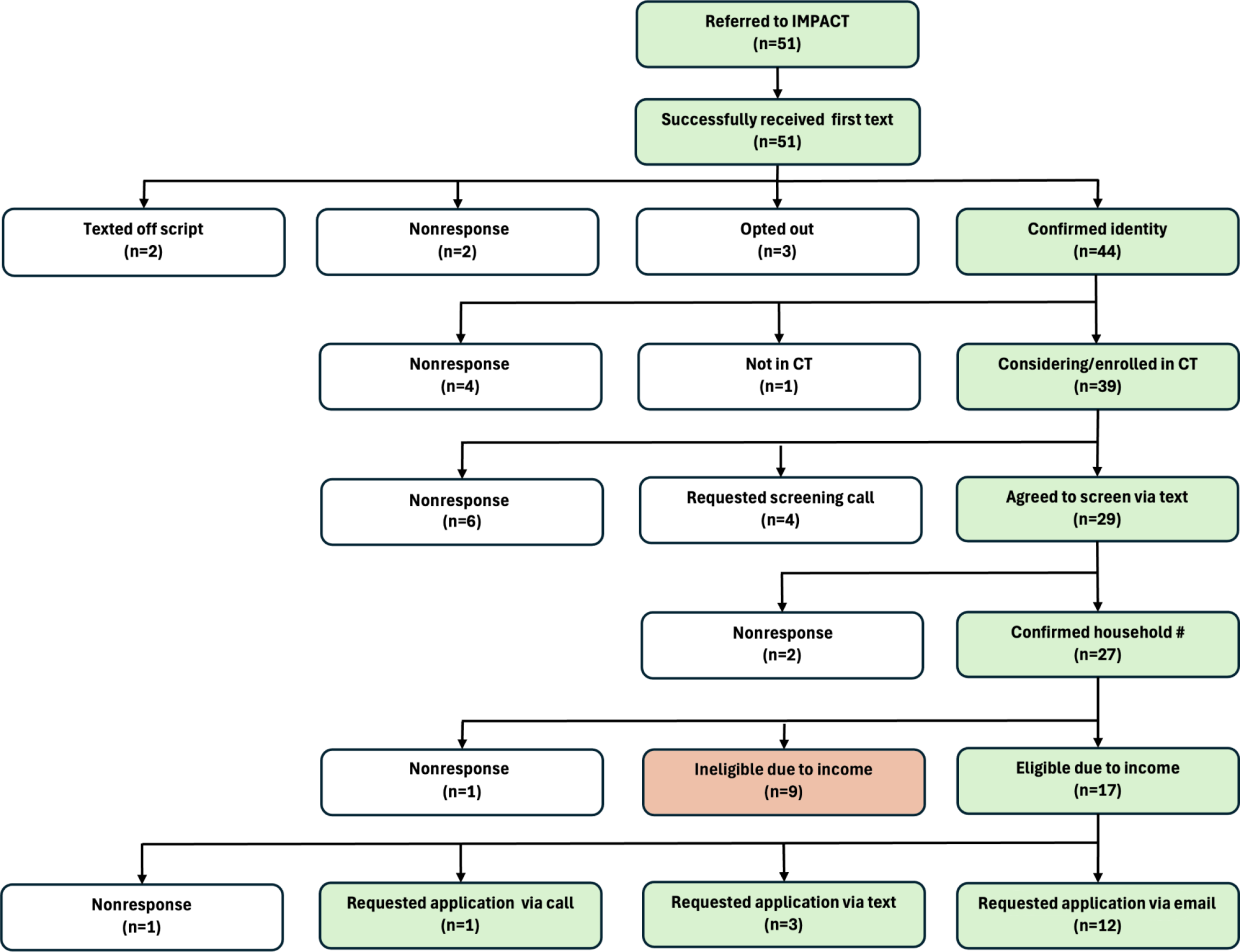
Patient engagement flow of financial reimbursement program automated text messaging program (n=51). CT: clinical trial; IMPACT: Improving Patient Access To Cancer Clinical Trial.

Of the 51 patients who received texts, 24 (47.1%) completed the entire text-based conversation, and 27 (52.9%) did not. As shown in Figure 2, a total of 33 (64.7%) participants agreed to screen for IMPACT eligibility (29 via text and 4 via phone call). Of those who were screened via text, 27 (52.9%) indicated their household size, and 26 (51%) went on to confirm their income level (17 qualifying and 9 disqualifying financially). Of the 17 eligible patients (33.3%), 12 (70.5%) requested an application via email, 3 (17.6%) requested via text, 1 (5.9%) did not respond, and 1 (5.9%) requested to complete the application via phone call.

**Figure 3.**
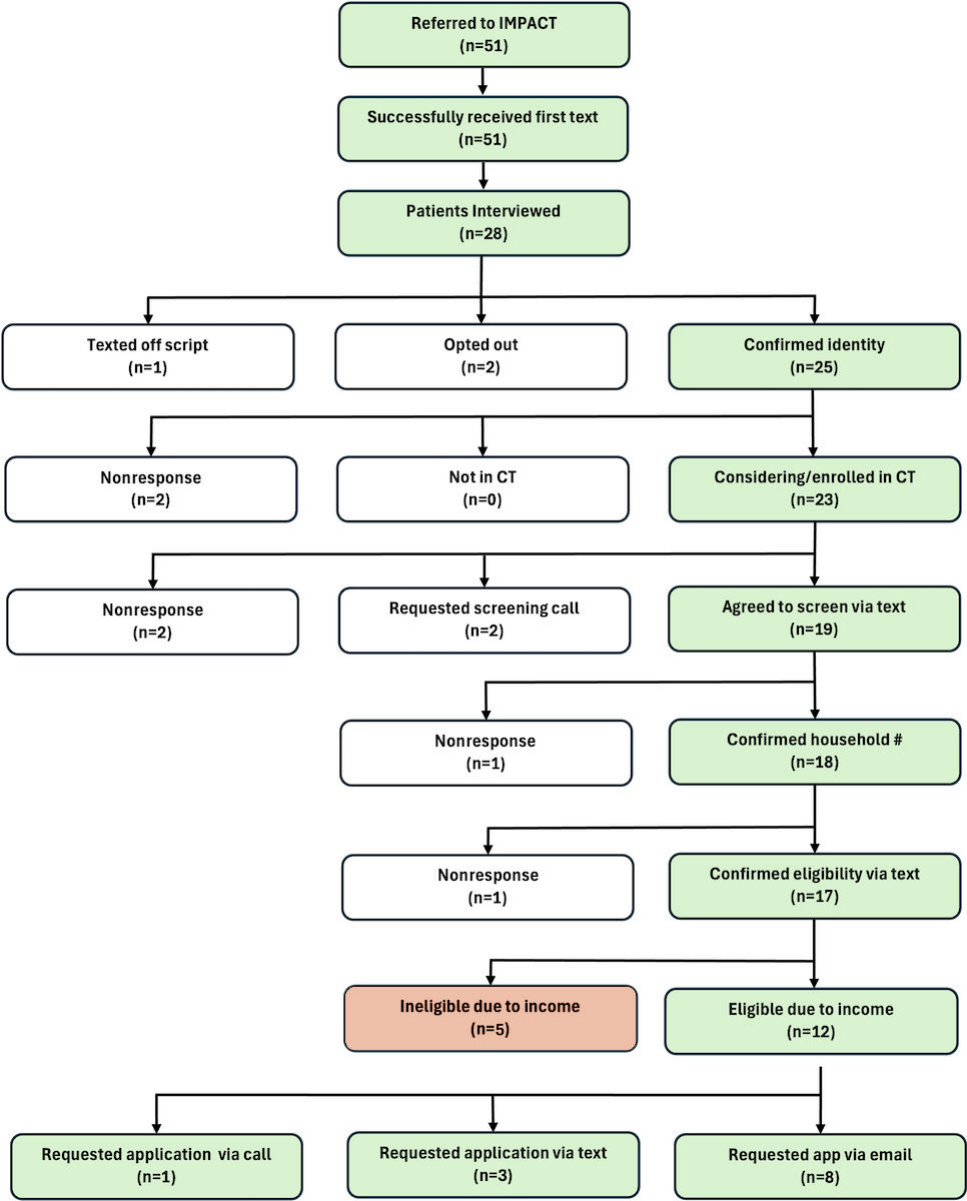
Interviewed patients' engagement flow of the financial reimbursement program automated text messaging program (n=28). CT: clinical trial; IMPACT: Improving Patient Access to Cancer Clinical Trial.

As shown in Figure 3, of the 28 patients who completed the interview, 17 (60.7%) completed the text-based conversation, and 11 (39.3%) did not. In the figure, these numbers can be obtained by adding all totals in white (11) and the final row totals (1 + 3 + 8) plus the 6 who were ineligible due to their income (17). A total of 21 participants (75%) agreed to screen for IMPACT eligibility (19 via text and 2 via phone call). Of those who screened via text, 18 (64.3%) indicated their household size, and 17 (60.7%) went on to confirm their income level (12 qualifying and 5 disqualifying financially). Of the 12 eligible patients (42.9%), 8 (66.7%) requested an application via email, 3 (25%) requested via text, and 1 (8.3%) requested to complete the application via phone call.

For both Figures2  3, the enrollment and screening numbers are time-bound. They reflect patient engagement and progression through the automated text-based FRP offering during the study period (March 26, 2024, to July 31, 2024).

#### Feasibility

Of the 51 patients who received the automated texts, 49 (96.1%) engaged with the texts. Four patients opted out and two patients texted off script. The remaining 46 (90.2%) patients consented to the automated texts, (Figure 2). Through the texts, we were able to screen 27 (52.9%) patients, and the enrollment rate among patients deemed eligible through text is 52.9% (9/17). Of those who enrolled after receiving an application via text, 33.3% (3/9) successfully enrolled in the FRP without any help from the IMPACT coordinator. The overall enrollment rate, including patients who received follow-up from the IMPACT coordinator via phone call, is 66.7% (16/24).

#### Technology Comfort

Interviewed patients were asked to respond on a scale of strongly disagree to strongly agree to the statement “I feel comfortable using technology such as a smartphone or computer to communicate with others.” Among the 28 patients who were interviewed, 78.6% (22/28) of patients responded “strongly agree,” 10.7% (3/28) of patients responded “somewhat agree,” and 10.7% (3/28) of patients were not asked this question (n=1 because version 1 of interview questions used and n=2 due to interviewer omission error). Version 2 of the interview was used for the remainder.

#### Satisfaction

Of the 51 patients who enrolled in the text messaging, 24 (47.1%) completed the NPS survey via text, and 11 (21.6%) completed the NPS survey during the semistructured interview. The overall NPS, calculated from the 35 patients who completed the NPS, regardless of method, is 51, indicating an “excellent” degree of patient satisfaction. The NPS, calculated from the 24 patients who completed the measure via text, is 54, indicating an “excellent” degree of patient satisfaction. The NPS, calculated from the 11 patients who completed the measure during their interview, is 45, indicating a “good” degree of patient satisfaction [15].

### Interview Results

A total of 28 interviews were conducted, lasting between 3 and 20 minutes. On average, patients completed the interview within approximately a month (range 0‐95 days) after the close of their text conversation.

### Clinical Trial Team Protocol Adherence (Awareness of the FRP)

Patient interview data revealed that only 46.4% (13/28) of patients were notified by the CT team about the IMPACT program and texts at the time of informed consent. Among the remaining patients, 14 (50%) learned about the IMPACT program at the time they received the first text, and 1 (3.5%) indicated they were unsure when they learned about the IMPACT program. As shown in Figure 1, of the 77 patients who consented to CCTs across the 3 research teams, only 51 (66%) patients were referred for the text conversation. Nearly one-third of eligible patients were not referred, which reflects a combination of system-level and staff workflow barriers, such as inconsistent protocol adherence.

### Barriers to and Facilitators of Completing the FRP Automated Text Messaging Program

#### Overview

Patient-reported barriers to and facilitators of completing the FRP automated text messaging are categorized in Table 2. We categorized the barriers according to system, individual, and implementation process, modeled after the Consolidated Framework for Implementation Research [17]. We have adapted this framework to use the following domains: system, individuals, and implementation process (where “system” represents a blend of the “inner settings” and “outer settings” domains).

**Table 2.** Patient-reported barriers to (n=28 interviews) and facilitators (n=28 interviews) of completing the financial reimbursement program automated text messaging program.

Code and theme (references/unique patients)				Example quote
System				
Barriers				
No prior mention of IMPACT^a^ by clinical trial team (3/3)				“I wasn’t aware that this was even a possibility...when I went through the consent, I discussed the trial with my doctor. They said I would not be compensated. I didn’t know there was an opportunity. So, I was surprised to get these texts. So, at first, I was skeptical...that this was real.”
Abundance of cancer center texts (3/2)				“You get a lot of things from [the cancer center] and it’s like if you want to be in this thing for after hospital, do you want to exercise? And a lot of them you say no to.”
Facilitators				
IMPACT coordinator help (17/14)				“As far as the text message, you’re the one I think I spoke to when I had a question about the number of people in the household, so just getting that cleared up right away.”
Prior mention of IMPACT or clinical trial team help (8/7)				“It helped that I already knew someone would be contacting me because if I wouldn't have known that up front, I may have thought it was a scam but since I already had a heads up, I had no problem participating via text.”
Coordination between CT^b^ team, IMPACT coordinator, and LCF^c^ (1/1)				“Well, my experience thus far has been seamless...with you with this group Lazarex and Penn, they've coordinated very well together.”
Individuals				
Barriers				
A lot going on (5/4)				“[A]t the time, I was worried about so many other things, I didn’t put necessarily the thought into this that I would have.”
Scam concerns (4/4)				“The concern will always be, with technology being as it is, sharing personal details...hacking...someone scheming your personal details.”
Uncomfortable sharing private information via text (2/2)				“[A]s soon as I read eligibility is based on your annual household gross income before taxes...I was no longer interested in really having that conversation, ‘cause I don’t like to disclose any of that information via text without talking to a human.”
Lack of foresight of financial toxicity (3/1)				“[W]hen I thought about it a little more, if I had been [traveling] and the study had continued, it would have been more of an expense than we originally realized.”
Tech savviness (2/1)				“The text program isn’t that difficult, but sometimes I get, like, stuck...I’m not technically savvy.”
Lack of awareness of text conversation time limit (1/1)				“I didn’t realize there was a [text conversation] time limit.”
Facilitators				
Age (1/1)				“Well, for my age bracket and for my, you know, constant use of the phone, to me, it's preferable to get a text”
Implementation process				
Barriers				
Limited information via text (4/4)				“...just the simplicity of it means more limited information.”
Confusion with income criteria outlined via text (3/3)				“I was confused because the way the question was worded.”
Abundance of information for the first time via text (1/1)				“It’s just a lot of information for the first time.”
Irreversibility of texts (1/1)				“I had put my income and I typed it too fast and was like, ‘oh, I need to combine my husband and my income per household’ then I was like, ‘Oh, that’s wrong. Oh, we might not qualify. I have to go back.’ And then that’s where it fell off.”
Texts more confusing or difficult than talking to a human being (1/1)				“Because I was all [confused] to begin with. So sometimes it’s just easier to talk to a human being.”
Facilitators				
Texts informative or clear (16/13)				“[I]t was pretty clear what you were asking for and the details of what might be covered.”
Text program easy (11/11)				“I would just say it was really easy through the text message.”
Multiple choice options for text replies (7/4)				“And they gave me the three choices, which was very easy...I like having the multiple choice.”
Texts convenient (4/3)				“I was...out of the office...so I was on the go [but] I was able to do this while moving around and not necessarily [have to] be on the phone.”
Texts concise (4/2)				“[Y]ou run the risk when you make a text too long that people just don't read it...so you got to be succinct and I think it was...just the right length.
Option to switch to call (3/3)				“I like the text because it gives you the option if you want to talk to someone, you can text assist and get a person, or [if you’re] comfortable with replying to the text messages, you can do it that way.”
Texts polite (2/2)				“Well just the text messages that I received were very polite, very nice.”
Timing of text relative to prior mention of IMPACT (1/1)				“[The text] came right away, pretty much within a week after me signing up for the clinical trial, signing the consent. So, it was still fresh in my mind. So I think that the timing was good.”

a IMPACT: Improving Patient Access to Cancer Clinical Trials.

b CT: clinical trial.

c LCF: Lazarex Cancer Foundation.

#### System Barriers

The most commonly cited system barrier was that patients were not systematically informed they would be receiving an IMPACT FRP text at the time of informed consent and were, therefore, skeptical of the texts. Particularly, some patients assumed it was sent to the wrong person, or in one case, a patient even deleted the text. One patient recounted:

*I guess when I first received it...I wasn’t fully aware of what it entailed. So...when I deleted it, you know...I don’t even remember deleting it*.

Patients also reported receiving texts from various groups at the cancer center, which led to their lack of engagement with the IMPACT FRP texts. One patient opted out of the text message and remarked:

*...I wasn’t quite sure what trial you were talking about, because it’s not delineated in the email or the text. So, I guess, having someone contact [me] to explain all that... you get a lot of things from Penn and it’s like, “if you want to be in this thing for after hospital,” “do you want to exercise?” And a lot of them you say no to.*

#### Individual Barriers

The most common individual barrier was the amount going on in patients’ lives. When asked why they did not respond at a specific point in the text conversation, 1 patient gave the following reason:

*Treatment...just a lot going on at the time. I think time-management with things going on...it’s just timing.*

Another patient responded:

*“I missed it, you know, I was in the hospital too.”*

Another significant individual barrier was the concern that the texts may have been a scam or could have led to hacking. When asked about concerns about sharing financial information via text, one patient mentioned the following:

*“[Y]ou know, hackers and stuff like that can get...information off your phone.*”

Other patients mentioned “making sure it wasn’t a scam” and “hacking*.*”

#### Implementation Process Barriers

Within the implementation process domain, significant barriers were related to the authenticity and clarity of the content of the text messages. Some patients mentioned that the texts should be more detailed. For example:

*I think you’d have to put a lot more detail in your text. And I don’t know if you have the ability to do all that, because I know sometimes, you’re limited. But there’s really no detail about me and about what clinical trial [you were referring to].*

The lack of information caused this patient to opt out of the text conversation. Another patient described the text introduction as “pretty vague” and mentioned the following:

*I think what would have helped me is...if [the texts] had a link...that I could have gone to [externally] to learn more about [the program]. And then...once I learned more about it, I probably would have felt more comfortable and confident in it*.

Patients also reported confusion with the income criteria outlined via text and how to determine their household size and income. One patient recalled:

*[I was confused] because the way the question was worded...it says, “how many people in your household, including yourself and all adults and dependents, claimed on your most recent tax return?” And so I put 4 because it’s my husband and I and our two adult children. But then [once I saw how high the income limit was]...the numbers seemed really high. So, then I was like, “I think it just means dependents.”*

#### System Facilitators

Patients consistently noted ‘help from the IMPACT coordinator’ as a facilitator. One patient described how the IMPACT coordinator facilitated the process:

*Even when I stopped, I felt like [I] confused myself with it, [the IMPACT coordinator] reached out and we were able to figure out whether I was eligible or not.”*

Another patient described receiving clarification about income criteria from the IMPACT coordinator:

*As far as the text message, you [the IMPACT coordinator] are the one I think I spoke to when I had a question about the number of people in the household, so just getting that cleared up right away.*

This particular patient texted in “ASSIST” after receiving the first screening text, which acts as a request to the IMPACT Coordinator to call the patient. The IMPACT coordinator reached out to this patient, and subsequently, the patient was able to continue enrolling in the FRP using the texts. Regarding their interaction with the IMPACT coordinator, this patient noted, “When I had a question, I got a phone call really quick, like way quicker than I expected.”

Many patients reported that their introduction to the IMPACT Program by their CT team (eg, clinical research coordinator or clinical research nurse) was a facilitator to completing the text conversation. Patients reported knowing “exactly what” the text conversation was because they “had been given some information prior.”

#### Individual Facilitators

Only 1 individual facilitator was reported. A single patient mentioned that their age bracket, combined with constant use of their phone, facilitated completion of the text conversation.

#### Implementation Process Facilitators

Many patients called the text messages “clear,” “straightforward,” “self-explanatory,” and “informative,” among others. Multiple patients described the text conversation as being “super,” “really,” or “very easy,” one specifically commenting they felt it was “easy for [them] to follow” and another mentioning they “thought it was easy to respond.”

### Advantages and Disadvantages of Texts

Finally, we asked patients what advantages and disadvantages they saw to offering the program through texts compared to offering the program through an IMPACT coordinator over a live phone call (Table 3). Approximately one-third of interviewed patients reported the text conversation as being quicker and more efficient than enrollment in the FRP by telephone call.

**Table 3.** Patient-reported advantages (n=28 interviews) and disadvantages (n=28 interviews) to offering the program through texts compared to offering the program through an IMPACT coordinator over a live phone call.

Code and theme (n references/n unique patients)^a^		Example quote
Disadvantages		
Lack of instant question clarification (5/5)		“If there’s confusion, it’s hard to address any confusion that might arise via text...so clarification questions...you could ask a person, but not a text system.”	
Requirement to share personal information via text (3/3)		“The disadvantage is that it’s not always within everybody’s comfort zone to provide a lot of information back and forth via text. Especially when it comes to personal, um, you know, financial information.”	
Texts more easily lost (2/2)		“Sometimes the person could possibly just delete the text. Whereas, the phone, they would have to pick up and you would at least get that initial engagement.”	
Confusion about purpose of texts if not received soon after informed consent (1/1)		“If [the timing between the mention of IMPACT by my CT team and reception of the first program text] had been longer, I would have been like, what is this about?”	
Advantages		
Easier (10/9)		“It’s always easier just to go through a text than...talking to somebody.”	
Quicker and more efficient (9/9)		“You know, you're not trading phone calls. You're not going through a long introduction; I think the advantage is because you read it and it’s quick. Most people read text messages real quick and glance through them.”	
More convenient (7/7)		“[With a] text message, you can just get to it when you have some time. Sometimes when you get a phone call, you’re not always available or sometimes you can’t talk at that moment.”	
Not required to talk to a person (6/5)		“Well, you know, sometimes...I don't want to be bothered talking to people on the phone for an hour.”	
Easier to discern if spam (4/4)		“A voice message, if you don't recognize the number...sometimes you just ignore it. Whereas a text message...shows you a couple lines...[it] started off with [my] name and [the cancer center name]. So...I would open that quicker than a long-distance number that I don't recognize.”	
More permanently available (3/3)		“I appreciate the text because there's the written text in front of me that I can respond to and I have documentation of.”	
More flexible (2/2)		“I wasn’t able to take a phone call at the time that I received that, but I could take a text...I might have been pinned in a waiting room somewhere because we have so many doctor’s appointments.”	
More widely used (2/2)		“People communicate with text all the time. So... it’s more of a natural thing these days.”	

a Of the reported disadvantages, some of the references were said as a hypothetical disadvantage rather than a disadvantage experienced while completing the text conversation. The hypothetical disadvantage counts are as follows: “lack of instant question clarification” (4/4), “requirement to share personal information via text” (1/1), “texts more easily lost” (2/2), and “confusion if text not sent soon after informed consent” (1/1).To determine the counts (n references/n unique patients) for patient-experienced disadvantages, subtract the hypothetical count (n references/n unique patients) from the table (n references/n unique patients) count.

## Discussion

### Principal Findings

This study showed that using an automated text messaging platform to offer, screen, and enroll patients in the IMPACT FRP is feasible and acceptable. To our knowledge, this is the first study to leverage text messaging to engage individuals in CCTs in an FRP. There was high engagement with texts (49/51, 96.1%), screening for the FRP (51%), FRP enrollment rate among patients deemed eligible through text (9/17, 52.9%), as well as overall enrollment rate (16/24, 66.7%), excellent degree of patient satisfaction (NPS=51), and high comfort with technology (78.6% strongly agreed to comfort with technology). In addition, one-third of all patients who received an application via text required no help from the IMPACT coordinator to complete the application. The use of the automated text messaging system significantly reduced the time spent contacting patients because initial engagement with the texts led to quicker responses from patients via telephone call. Moreover, almost one-third of interviewed patients reported the text conversation as being quicker and more efficient than enrollment in the FRP by telephone call.

We identified barriers and facilitators at the system, individual, and implementation level that could inform program designs to increase participation. The most frequently cited system facilitator was the assistance provided by the IMPACT coordinator, highlighting the importance of having a knowledgeable and responsive contact to complete enrollment for those who opt out of texts and address questions that arise. Another facilitator included communication from the CT teams regarding the texts, which led to a decrease in skepticism and an increase in the likelihood of response to the text messages. Conversely, the most cited system barrier was related to the lack of communication from the CT team. Although CT teams were asked to notify patients of the FRP texts, as well as hand out our study flyer, only 46.4% (13/28) of interview patients were notified by the CT team that they would receive FRP texts, highlighting the persistence of a barrier from IMPACT 1.0: lack of patient awareness of the FRP due to inconsistent mentions from CT team members. Moreover, approximately one-third of all patients potentially eligible for the FRP were not referred to the IMPACT coordinator for enrollment in the automated texts, and thus, this is an important barrier to the implementation of this program.

One possible way to ensure that patients are informed by their teams about the FRP texts is to include referral to the program as part of the standard operating procedures in protocols and informed consent forms to ensure all patients are notified of the program and will anticipate receiving the first text. This strategy may also reduce text authenticity concerns, which were a significant patient-reported barrier within the individual domain. In addition, inclusion of the program website link or requiring a 2-factor authentication or a unique patient identifier in the initial text could help increase the legitimacy of the text messages.

Another barrier to implementation is related to the most cited barrier: “limited information via text.” Owing to the Health Insurance Portability and Accountability Act laws, the texts could not contain personal health information and, therefore, could not mention CT information or even the word “cancer.” Omission of this information caused at least one patient to opt out of the text conversation. To address this issue, it may be helpful to create a text introduction with a layered information approach. Through this approach, basic program information would be included via text while also including one or multiple URL links, which navigate a patient to a more detailed resource or website. In addition, we plan to address language barriers in future deployments of the automated text system by integrating multilingual capabilities. This includes developing and implementing message libraries in the most common languages spoken by the target patient population, starting with Spanish but expanding to others as identified through community needs assessment.

The prior literature has demonstrated that text-based communication can be used in lieu of clinic visits for symptom monitoring, which can then reduce clinic wait time before chemotherapy [18] and increase access to supportive care (such as physical activity motivation and medication reminders to promote adherence). A systematic review demonstrated that text-based messaging improved the patient experience, but outcomes were mixed in part because of the heterogeneity in study design, measured outcomes, and threat of bias [19]. This study adds to the body of literature showing that text messaging is an acceptable way to communicate and engage patients in a variety of health-related behaviors. Text-based communication is more accessible to patients than online electronic medical record portals: 97% of Americans state they communicate via text, whereas only 65% of Americans who were offered an online patient portal accessed it [20].

The limitations of this study include a relatively small sample size, with participants from a single institution and conducted with English-speaking populations, which may reduce the generalizability. The demographic composition of the sample, while diverse, may not fully represent all patient populations with cancer. The interview process may have been subject to recall bias as well, as some participants were interviewed weeks after their text interaction. Finally, participants with low technology literacy may not have responded to the texts, introducing the possibility of selection bias.

### Conclusions

Despite these limitations, the study is among the first to introduce and evaluate a text-messaging system to introduce, screen, and enroll patients in an FRP. The findings demonstrate digital platforms such as W2H can be leveraged to develop automated text messages that increase awareness, screen, and enroll patients in FRPs. W2H can be deployed at other institutions, and thus, this program can be scaled. Automated text-based messaging provides a potential low-cost opportunity to scale the program to other institutions. Future directions include conducting cost-effectiveness analysis to inform dissemination, implementation, and policy.

## Supplementary material

10.2196/78916 Multimedia Appendix 1 Text messages and study protocol.

10.2196/78916 Multimedia Appendix 2 IMPACT eligibility criteria and process.

10.2196/78916 Multimedia Appendix 3 In-depth interview guide.

## Abbreviations

CAR-T: chimeric antigen receptor T cell
CCT: cancer clinical trial
CT: clinical trial
FRP: financial reimbursement program
FT: financial toxicity
IMPACT: Improving Patient Access to Cancer Clinical Trials
NPS: Net Promoter Score
OOP: out-of-pocket
W2H: Way To Health
