# Supplementary material for: Feasibility and Acceptability of Automated Texts to Offer, Screen, and Enroll Patients in a Cancer Clinical Trial Financial Reimbursement Program: Mixed Methods Study
Source: JMIR Form Res. 2026 Jul 9;10:e78916. doi: 10.2196/78916 (PMC13348992; doi:10.2196/78916)
Supplement: Multimedia Appendix 1 [file formative-v10-e78916-s001.docx]

**Appendix 1: Text Messages & Study Protocol**

**
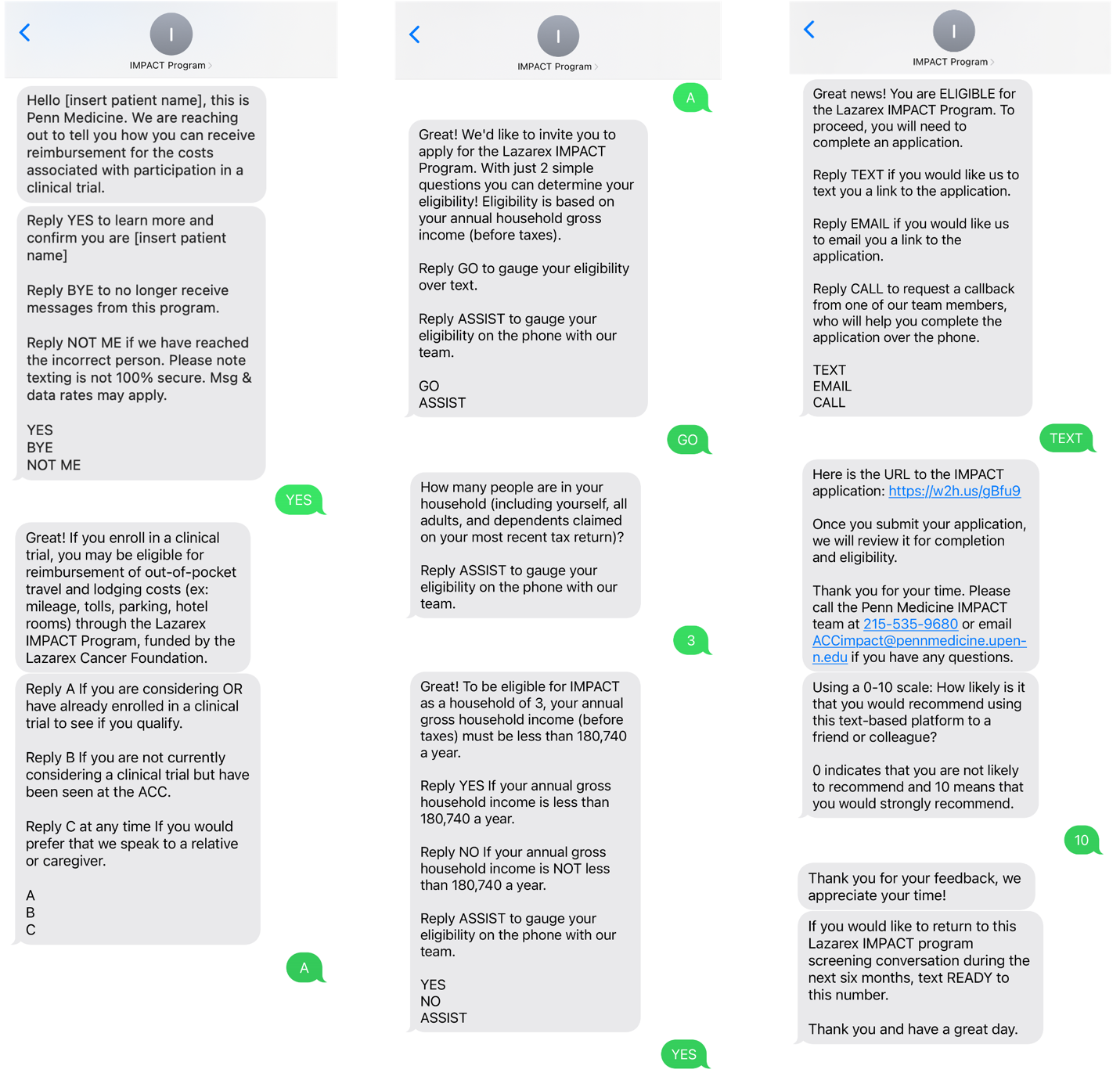
**

Although not pictured in the exemplar texts, the text conversation included reminder messages for patients who did not engage with texts. Three automated reminders were put in place: one after the first non-response at 5PM the following day, one after the second non-response at 9AM the following day, and one after the third non-response at 5PM the following day. After the fourth non-response, patients receive a message letting them know the conversation had closed but that they can type “READY,” within the next 6 months, to re-open the conversation. Patients who successfully received an application for the IMPACT program, but did not complete the application after one week, also received a manual text message reminder through the same conversation, manually generated by the IMPACT coordinator, which reminded them to complete their application. The end of a text conversation was signaled by non-response after 3 reminders or completion of the NPS survey.
