# Supplementary material for: Feasibility and Acceptability of Automated Texts to Offer, Screen, and Enroll Patients in a Cancer Clinical Trial Financial Reimbursement Program: Mixed Methods Study
Source: JMIR Form Res. 2026 Jul 9;10:e78916. doi: 10.2196/78916 (PMC13348992; doi:10.2196/78916)
Supplement: Multimedia Appendix 2 [file formative-v10-e78916-s002.docx]

**Appendix 2: IMPACT Eligibility Criteria**

Patients were eligible for the IMPACT Program if: (1) they were enrolled in a cancer therapeutic clinical trial and (2) their household income was less than 700% of the most recent U.S. Department of Health and Human Services (HHS) Poverty Guidelines; for example, in 2024, a household of 3 could have an annual income could up to $180,740 to qualify for 50%, $142,010 to qualify for 75%, and $103,280 to qualify for 100% reimbursement of OOP travel costs incurred.

If eligible, patients could choose to receive a program application via three different methods: text, email, and call. If a patient chose text or email, they would be sent a URL web link through each respective method. The URL web link brought patients to an online fillable application that was completed and submitted directly on the browser. If a patient chose call, the IMPACT coordinator would call the patient to complete the application via telephone. The program application requires household income verification (also known as proof of income (POI)).

Acceptable forms of POI include submission of the first two pages of the signed copy of the household’s most recent income tax return or, if they do not file a tax return, copies of their most recent pay stub, unemployment check, or SSI, SSD, or public assistance benefit notification. If a patient or household is not currently employed, the Lazarex Cancer Foundation accepts a signed letter from the patient stating their current financial situation.

Patients who chose to receive their application link via text or call were responsible for submitting their own POI. Patients who completed the application via telephone with the IMPACT coordinator sent their POI to the IMPACT coordinator by email or directly to LCF via fax or mail. Upon receipt of the application and income verification by LCF, patients received notification of approval or denial of their application within 7-14 days. The date of approval serves as the first date for allowable expense submission, which requires submission of receipts and a travel log. The Lazarex travel log is a PDF document that patients must complete, once monthly, detailing their travel and lodging expenses for the previous medical month. On the travel log, patients are provided with space to detail their expenses, broken down by lodging, flights, mileage, parking, tolls, and ground transportation (ride share company, public transportation, taxi, etc.).
