# Supplementary material for: Feasibility and Acceptability of Automated Texts to Offer, Screen, and Enroll Patients in a Cancer Clinical Trial Financial Reimbursement Program: Mixed Methods Study
Source: JMIR Form Res. 2026 Jul 9;10:e78916. doi: 10.2196/78916 (PMC13348992; doi:10.2196/78916)
Supplement: Multimedia Appendix 3 [file formative-v10-e78916-s003.docx]

**Appendix C: In-Depth Interview Guide**

Interviews were completed with the primary individual who engaged with the automated texts. Thus, prior to the interview, we noted if the interviewee was the patient, spouse, child, caregiver, or other. The interview questions focused on their experience, the barriers and facilitators of learning about, screening for, and enrolling in the IMPACT Program, and suggestions for improving the process. After completing the first patient interview, the interview questions were adapted to probe for more in depth responses and to encompass more questions. All interviewees, except the first participant, were asked the second version of interview questions. Below are both versions of the full interview questions.

Interview Questions – Revised

The in depth, semi-structured interview will consist of a variety of the following questions (depending on the last text to which a patient responded):

1. *Are you able to pull up the text conversation you had regarding the Lazarex IMPACT Program so that you can reference it during this interview? (the phone number is (215)-709-6485 or you can search your text messages for a key word - e.g. Lazarex)*
2. *When did you learn about the Lazarex IMPACT program?*
   - 1. *Was it at the time of informed consent?*
     2. *Was it before receiving the texts about the program?*
        1. *If yes, how did you learn about the Lazarex IMPACT program.*
3. *Did you opt out of the texts (reply BYE) at any point?*
   1. *If yes, what factors led you to reply BYE?*
      1. *Do you have any suggestions on how to improve our communication via text?*
   2. *If No to question 2, Did you request a callback (reply CALL) at any point?*
      1. *If yes, what factors led you to request a callback?*
4. *Do you have any suggestions on how to improve our communication via text?*
5. *Based on where your conversation ended, I’d like to ask your opinion on the effectiveness of the components of the conversation (introduction, screening, and enrollment).*
   1. *If made it through introduction, Did you think that a text-based introduction to the IMPACT program was effective?*
      1. *Why or why not?*
   2. *If made it through screening, Did you think that a text-based screening for the IMPACT program was effective?*
      1. *Why or why not?*
   3. *If it made it through enrollment, Did you think that a text-based enrollment for the IMPACT program (which makes the application available in three ways – online, pdf for completion in writing, and completion via phone) is effective?*
      1. *Why or why not?*
6. *As you may remember, the text messages offered three options for enrollment in the IMPACT program (online link via text, online link via email, phone call). What factors did you consider in choosing a method to enroll in the IMPACT program – options 1, 2, or 3 (receiving online application via text vs. are receiving online application via email vs. completing the application via telephone call with a research coordinator)?*
   1. *Other language: why did you chose the method of enrollment/application completion you did?*
7. *Do you see any advantages to offering the program through texts compared to offering the program through a research coordinator? Disadvantages?*
8. *What barriers are there to enrolling in the IMPACT program via texts?*

*What Facilitators are there to enrolling in the IMPACT program via texts?*

1. *Do you have any additional comments regarding the or platform (Way to Health) in your involvement enrolling in the IMPACT program?*

Interview Questions – Original

The in depth, semi-structured interview will consist of a variety of the following questions (depending on the last text to which a patient responded):

1. *Are you able to pull up the text conversation you had regarding the Lazarex IMPACT Program so that you can reference it during this interview? (the phone number is (215)-709-6485 or you can search your text messages for a key word - e.g. Lazarex)*
2. *When did you learn about the Lazarex IMPACT program?*
   - 1. *Was it before receiving the texts about the program?*
        1. *Was it before receiving the text messages?*
        2. *Was it at the time of informed consent for your clinical trial?*
3. *(If learned about it before receiving texts) How did you learn about the IMPACT program?*
   1. *Word of mouth?*
      1. *Who mentioned it to you?*
         1. *Study coordinator, physician, etc.?*
      2. *Online*
         1. *Where online did you find it?*
4. *(If patient opted out of the texts (reply BYE) at any point) What factors led you to reply BYE?*
   1. *What suggestions do you have to help us communicate with you via text?*
5. *(If patient requested a callback (reply CALL) at any point) What factors led you to request a callback?*
6. *(If patient did not choose a method to enroll) What factors led you to not choose a method to receive the IMPACT application?*

*Based on where your conversation ended, I’d like to ask you a few questions regarding the 3 main components of the conversation: introduction, screening, and enrollment in the IMPACT program.*

1. *(If made it through introduction), The introduction is comprised of the first 5 texts in which the program was described to you. Is there any other information you would have liked to receive in the introduction texts?*
2. *(If made it through screening) The screening consisted of the next 3 texts. The first text asked if you would like to move onto screening. This text was followed by 2 screening questions, the first regarding the number of people in your household and the second asking if your income falls above or below the program income criteria.*
   1. *(Now that we’ve reminded you about the screening portion of the texts, we’re going to read a statement to you and ask that you respond on a scale of…) I felt comfortable confirming if my household income fell above or below the IMPACT program’s financial eligibility criteria via text.*
      1. *Strongly agree, somewhat agree, neither agree nor disagree, somewhat disagree, strongly disagree.*
   2. *(What concerns, if any, did you have about sharing financial information with Penn Medicine via text?*
3. *(If made it through enrollment) The final portion of the texts allows you to choose a method for receiving the enrollment application (either via text or email) which allows you to complete the application via a fillable pdf online. Alternatively, you may have chosen to complete the application over the phone with an IMPACT team member.*
   1. *Which method did you choose to receive your enrollment application (if patient doesn’t remember and cannot look at their texts, W2H has access to this info)*
   2. *What factors did you consider in choosing a method to enroll in the IMPACT program – options 1, 2, or 3 (receiving online application via text vs. are receiving online application via email vs. completing the application via telephone call with a research coordinator)?*
      1. *Other language: why did you choose the method of enrollment/application completion that you did?*
4. *What advantages do you see to offering the program through texts compared to offering the program through an IMPACT team member over a live phone call? Disadvantages?*
5. *(for patients who received application) What barriers made it difficult to learn about, screen for, and receive an application for the IMPACT program via text? (for patients who did not request application) What barriers prevented you from learning about, screening for, and receiving and application for the IMPACT program via text?*
   1. *(If patient let conversation close before NPS) What factors prevented you from completing this text message conversation (which aimed to introduce you for the program, screen you for the program, and provide you with an application to enroll in the IMPACT program)?*
   2. *(For patients who received the application link, but never enrolled themselves in IMPACT) What could have been different about this process that would have increased the likelihood of you submitting your IMPACT application? (Alternate wording if patient needs more prompting: What information, if any, would you have liked us to include in the text messages to prepare you for completion of the Lazarex IMPACT application?)*
6. *What facilitators made it easy to learn about, screen for, and receive an application for the IMPACT program via text?*
   1. *(If patient needs more prompting or doesn’t understand question) What factors helped you complete this text message conversation (which aimed to introduce you for the program, screen you for the program, and provide you with an application to enroll in the IMPACT program)?*
7. *I feel comfortable using technology such as a smart phone or computer to communicate with others.*
   1. *Strongly agree, somewhat agree, neither agree nor disagree, somewhat disagree, strongly disagree*
8. *What additional comments do you have regarding the process of learning about, screening for, and receiving an application through a text-based platform (Way to Health) and your experience enrolling in the IMPACT program?*
9. (If patient does not complete NPS over text) “Using a 0-10 scale: How likely is it that you would recommend using this text-based platform to a friend or colleague?”
